# Selectivity of the botanical compounds to the pollinators *Apis mellifera* and *Trigona hyalinata* (Hymenoptera: Apidae)

**DOI:** 10.1038/s41598-020-61469-2

**Published:** 2020-03-16

**Authors:** Isabel Moreira da Silva, José Cola Zanuncio, Bruno Pandelo Brügger, Marcus Alvarenga Soares, Antônio José Vinha Zanuncio, Carlos Frederico Wilcken, Wagner de Souza Tavares, José Eduardo Serrão, Carlos Sigueyuki Sediyama

**Affiliations:** 1 Departamento de Fitotecnia, Universidade Federal de Viçosa, 36570-900 Viçosa, Minas Gerais Brasil; 2 Departamento de Entomologia/BIOAGRO, Universidade Federal de Viçosa, 36570-900 Viçosa, Minas Gerais Brasil; 3 Programa de Pós-Graduação em Produção Vegetal, Universidade Federal dos Vales Jequitinhonha e Mucuri, 39100-000 Diamantina, Minas Gerais Brasil; 4 Departamento de Engenharia Florestal, Universidade Federal de Viçosa, 36570-900 Viçosa, Minas Gerais Brasil; 5 Departamento de Proteção Vegetal, Universidade Estadual Paulista, Botucatu, São Paulo Brasil; 6 Asia Pacific Resources International Holdings Limited, Riau Andalan Pulp and Paper, Pangkalan Kerinci, Riau 28300 Indonesia; 7 Departamento de Biologia Geral, Universidade Federal de Viçosa, 36570-900 Viçosa, Minas Gerais Brasil

**Keywords:** Agroecology, Animal behaviour

## Abstract

The toxicity of essential oils that can be used in insect pest management to pollinators needs further studies. *Apis mellifera* Linnaeus and *Trigona hyalinata* (Lepeletier) (Hymenoptera: Apidae) foragers were exposed by three pathways to ginger, mint, oregano and thyme essential oils to provide their LC_50,_ LD_50_ and LC_90,_ LD_90_. Oregano and thyme were more toxic through contact and topically for *A*. *mellifera* while the toxicity of mint and ginger was lower. *Trigona hyalinata* was more tolerant to the essential oils than *A*. *mellifera*. In the walking test, the area was treated (totally or partially) with sub-doses (LC_50_) obtained via contact. The area fully treated with oregano reduced the distance traveled and the movement speed increased the number of stops by *A*. *mellifera*. Similar results were observed for *T*. *hyalinata* with oregano and thyme oils. *Apis mellifera* showed irritability remaining shorter time in the area partially treated with ginger, mint and thyme essential oils while *T*. *hyalinata* had similar behavior with ginger and thyme. Essential oils did not repel *A*. *mellifera* or *T*. *hyalinata*, but those of ginger, mint and thyme reduced the time spent by *A*. *mellifera* in areas treated with sublethal doses. Oregano and thyme essential oils reduced the survival, mainly, of *A*. *mellifera*, while ginger and mint were selective for both pollinators.

## Introduction

Bees are essential for plant propagation^1^, but factors such as pathogens, habitat losses and intensive pesticide use are reducing their populations^2^. The decline of bee colonies, known as Colony Collapse Disorder (CCD), threatens pollination and the production of honey, propolis, royal jelly, and wax^3,4^.

Bees are exposed to insecticides during the pollen and nectar harvesting through contact with the treated plant surface and the ingestion of sap from seeds coated with systemic insecticides^5,6^ abamectin, acetamiprid, cartap-hydrochloride, chlorfenapyr, deltamethrin and thiamethoxam were toxic to *Apis* *mellifera* Linnaeus, 1758 (Hymenoptera: Apidae) workers by direct spraying; diet treated and contact with pulverized leaves^2^. Larvae mortality was high and adult mobility of *Melipona quadrifasciata* Lepeletier, 1836 (Hymenoptera: Apidae) low with diet treated with imidacloprid^7^. These insecticides act on arthropods, causing physiological and behavioral effects by directly interfering in the acetylcholine receptors^7^. In addition, insecticides may affect learning, foraging, growth, besides pupa malformation, adult emergence and reproduction^8,9^ and caused irritability and repelled bees^7^. Residues in wax, nectar and pollen reduce the quality and value of these products^4^.

The vulnerability of *A*. *mellifera* to pesticides has been studied^2,10^ but the effect of these products on native stingless bees such as *Trigona hyalinata* (Lepeletier) (Hymenoptera: Apidae) is little understood. The development period of stingless bees is generally longer than that of *Apis* spp., and, therefore, their survival is more under threat, besides being native and important in agrosystems^11^. Behavioral mechanisms, such as escape after the detection of irritants or repellents, may reduce the contact of insects with toxic substances^7,12^.

Botanical insecticides, used in pest control^13–15^ have advantages such as efficiency in herbivore control, slow induction of insect resistance due the complexity of compounds, lower toxicity to non-target organisms and lower environmental impact given their faster breakdown and volatilization, minimizing the residual contact with pollinators and biological control agents^16,17^ compared to synthetic chemicals^18^. However, plant substances may repel or are toxic to bees and natural enemies^19–21^ and therefore require toxicological evaluation.

The essential oils effects of ginger, mint, oregano and thyme plants and their major compounds control several insect pest^14,16,19,22,23^. However, their possible effects (i.e., toxicity, behavior) on bees were not investigated.

The objective was to determine the lethality (LC_50,_ LD_50_ and LC_90,_ LD_90_) of ginger, mint, oregano and thyme essential oils used for pest management, and the walking patterns of *A*. *mellifera* and *T*. *hyalinata* pollinators in areas treated totally or partially with the LC_50_ of these botanical products.

## Material and methods

### Bee collection and preparation of the essential oil concentrations

*Apis mellifera* and *T*. *hyalinata* forager workers were collected in colonies from the Apiary of the Federal University of Viçosa in Viçosa, Minas Gerais state, Brazil using a large mouth vial at their entrance. The tests were conducted in B.O.D. at 32 ± 1 °C and 28 ± 1 °C for *A*. *mellifera* and *T*. *hyalinata*, respectively.

Ginger, mint, oregano and thyme essential oils produced by steam distillation of leaves (mint and oregano), leaves-flowers (thyme) and roots (ginger) were acquired from an industrial supplier. Chromatography of the essential oils was provided by the company (Table 1).

**Table 1:** Common name (Common), scientific name (Sci.) and family, major components and part of the plant used to extract the essential oils tested on *Apis mellifera* and *Trigona hyalinata* (Hymenoptera: Apidae).

Common	Sci. (Family)	Major components (%)	Part
Ginger	*Zingiber officinale* (Zingiberaceae)	zingiberene (33%), beta-sesquipelandene (12%), β-bisabolene (10%), camphene (8%), myrcene (7%)	Root
Mint	*Mentha piperita* (Labiatae)	menthol (55%), menthone (25%), menthyl acetate (10%)	Leaf
Oregano	*Origanum vulgare* (Lamiaceae)	carvacrol (70%), p-cymene (15%), thymol (4.3%)	Leaf
Thyme	*Thymus vulgaris* (Lamiaceae)	thymol (50%), p-cymene (40%), linalool (6.0%)	Leaf/Flower

### Toxicity bioassay

The concentrations of the insecticides, established in preliminary tests, were used to obtain the mortality rate of *A*. *mellifera* and *T*. *hyalinata*. The concentrations of the ginger, mint, oregano and thyme oils were 0.5 to 25% (v/v), varying with exposure and contact mode. Acetone was used as a solvent, as it did not present toxicity to bees in preliminary tests with water and pure acetone. The bees were placed in a refrigerator for five minutes to facilitate their handling before mounting the experiments.

#### Exposure by contact (surface treated)

Filter paper pieces (9 cm diameter) were soaked in 500 μL of each oil solution and left to dry in the shade for one hour. Ten bees were placed per plastic pot (250 ml) lined with the treated filter paper. These pots were punctured on the side, to insert a tube containing water and honey (1:1), and the caps perforated and covered by organza for ventilation. A cotton swab, soaked with water, was placed at the bottom of the pots. Bee mortality was evaluated after 24 hours^24^.

#### Topical exposure

Bees received, topically, 1 μL of the pre-established concentrations of the oils in the mesonotum between the first and second pairs of legs. Ten bees were placed per plastic pot (250 mL) with the same application conditions via contact. Mortality was assessed after 24 hours^25^.

#### Exposure by ingestion

The bees were deprived of food for thirty minutes before offering the essential oils. The essential oils were incorporated into the liquid diet (water: honey) with the Tween^®^ 80 emulsifier, 0.01% (v/v), for solubilization. Ten bees were placed per plastic pot (250 mL) and water provided in all treatments. Mortality was evaluated after 24 hours^26^.

### Walking bioassays with bees

*A*. *mellifera* and *T*. *hyalinata* in areas totally or partially treated with ginger, mint, oregano and thyme essential oils were evaluated in two bioassays^27^. These areas were assembled using Petri dishes (9 cm diameter) with the bottom lined with filter paper, the walls coated with Teflon^®^ PTFE (DuPont, Wilmington, USA) and covered with clear plastic film to avoid insect escape. The filter paper was treated with 500 μL of the essential oils in the LC_50_ obtained in the contact test. The control had only acetone on the paper.

The bioassay, without choice, had two areas, the first fully treated with the essential oil and the other with acetone. The distance traveled, number of stops and walking speed of the bees were evaluated. In the bioassay with choice, one area was half-treated with the essential oil and the other was not. The time spent in each area was evaluated.

In both bioassays, the areas were recorded using a video camera tracking system coupled to a computer (ViewPoint Life Sciences Inc., Montreal, Canada) with a bee released in the center and its movement evaluated for 10 minutes.

### Statistical analysis

The toxicity bioassay was developed in a completely randomized experimental design (DIC). The treatments were represented by the concentrations of the oils and the control, with five replications, each plot with 10 bees. The data were corrected by Abbott’s formula^28^. Concentration-mortality curves were estimated by Probit analysis and lethal concentrations or dose (LC_50_ or LD_50_ and LC_90_ or LD_90_) and their confidence limits determined. The relative tolerance indices  between species (RTI_50_ = LC_50_ or LD_50_ of the product for *T*. *hyalinata*/LD_50_ or LD_50_ for *A*. *mellifera*) was calculated.

The walking test was conducted in DIC with 14 replications, each with a bee. The filter paper and the bees were replaced at each replication. The data was submitted to variance analysis and the means compared by the Tukey test at 5% for the bioassay without choice and the t-test at 5% for the one with choice.

## Results

### Toxicity bioassay

The toxicity of the ginger, mint, oregano and thyme essential oils varied according to the application mode and rates as well as to bee species. The essential oil toxicity was, in descending order, oregano, thyme, mint and ginger for *A*. *mellifera* and *T*. *hyalinata* after 24 h (Tables 2 and 3).

**Table 2:** Toxicity of ginger, mint, oregano and thyme essential oils to *Apis mellifera* and *Trigona hyalinata* (Hymenoptera: Apidae) by exposure via contact for 24 h.

Treat.	Species	N	LC_50_ (IC) %	LC_90_ (IC) %	Slope	*Chi2*
Ginger	*A*. *mellifera*	400	22.01(20.89–23.01)	26.53 (25.22–28.56)	15.80 ± 2.23	50.22
	*hyalinata*	400	24.17 (22.36–25.88)	38.01 (34.12–45.42)	6.52 ± 0.94	47.28
Mint	*A*. *mellifera*	350	13.35 (12.21–14.28)	17.24 (16.03–19.23)	11.52 ± 1.87	38.00
	*T*. *hyalinata*	400	21.61 (20.20–22.96)	30.74 (28.20–35.29)	8.37 ± 1.19	49.81
Oregano	*A*. *mellifera*	300	0.95 (0.71–1.24)	3.22 (2.31–5.31)	2.42 ± 0.33	53.00
	*T*. *hyalinata*	300	7.14 (6.08–8.13)	10.87 (9.47–13.31)	7.00 ± 1.08	41.72
Thyme	*A*. *mellifera*	300	2.61(2.05–3.15)	6.39 (5.16–8.75)	3.30 ± 0.47	48.50
	*T*. *hyalinata*	250	8.29 (6.90–9.51)	18.15 (15.29–23.81)	3.76 ± 0.56	45.24

Treat = Treatment, N = individuals number, IC = Confidence interval of 95%, Chi2 = chi-squared. Significance level of P < 0.0001.

**Table 3:** Toxicity of the ginger, mint, thyme and oregano essential oils to *Apis mellifera* and *Trigona hyalinata* (Hymenoptera: Apidae) by exposure via topically by 24 h.

Treat.	Species	N	LD_50_ (IC) %	LD_90_ (IC) %	Slope	*Chi2*
Ginger	*A*. *mellifera*	300	17.98 (15.09–19.68)	27.41 (24.73–34.18)	7.00 ± 1.57	19.84
	*T. hyalinata*	400	32.65(29.26–37.14)	77.22 (61.86–109.66)	3.42 ± 0.43	63.90
Mint	*A*. *mellifera*	300	12.58 (8.22–14.62)	25.50 (21.14–46.44)	4.17 ± 1.20	11.73
	*T*. *hyalinata*	400	16.38 (14.21–18.26)	35.97 (30.82–45.95)	3.75 ± 0.51	52.71
Oregano	*A*. *mellifera*	300	2.03 (1.32–2.87)	9.88 (6.47–19.61)	1.86 ± 0.30	39.14
	*T*. *hyalinata*	300	4.57 (3.37–5.79)	20.01 (15.04–30.32)	1.63 ± 0.17	61.05
Thyme	*A*. *mellifera*	300	3.30 (2.13–4.38)	9.84 (7.54–14.36)	2.50 ± 0.36	47.62
	*T*. *hyalinata*	300	6.53 (4.91–8.12)	30.41 (22.82–47.06)	1.92 ± 0.25	58.22

Treat= Treatment, N = individuals number, IC = Confidence interval of 95%,

*Chi2* = chi-squared. Significance level of P < 0.0001.

*Trigona hyalinata* was more tolerant (ITR_50_ > 1.0) to ginger, mint, oregano and thyme than *A*. *mellifera* after 24 h by application on contaminated (topical) and topical surfaces (Table 4).

**Table 4:** Relative tolerance indices (RTI_50_) of the ginger, mint, oregano and thyme essential oils for *Apis mellifera* and *Trigona hyalinata* (Hymenoptera: Apidae) adults.

Essential oils	RTI_50_ (Th./Am.)	
	Contact Topic	
Ginger	1.10	1.82
Mint	1.62	1.30
Oregano	7.52	2.25
Thyme	3.18	1.98

Relative tolerance index [RTI50 = LC50 (contact) or LD_50_ (topical)] for *T. hyalinata*/LC50 (contact) or LD_50_ (topical)] for *A. mellifera*). Th./Am = *T. hyalinata*/*A. mellifera*.

There was a more homogeneous response by *A*. *mellifera* and *T*. *hyalinata* bees to dosage variability of the ginger, mint, oregano and thyme oils by contact as shown by the greater slope (slope) of the concentration-mortality curve (Tables 2 and 3).

The oregano oil was most toxic, mainly to *A*. *mellifera*. This product was more toxic than ginger at 23.17x by contact and 8.86x by topically for *A*. *mellifera*. Oregano was more toxic to *T*. *hyalinata* than ginger at 3.39 x per contact and 7.14x per topically (Tables 2 and 3).

Thyme oil toxicity was intermediate while those of ginger, mint and oregano, with 8.43x per contact and 5.45x by topically was more toxic than ginger for *A*. *mellifera*. Thyme was more toxic to *T*. *hyalinata* than ginger at 2.92x(contact) and 5.00 x(topical) (Tables 2 and 3).

Mint and ginger oils were the most selective for *A*. *mellifera* and *T*. *hyalinata* with higher values in lethal concentrations or dose.

The high mortality of *A*. *mellifera* and *T*. *hyalinata*, by ingestion with all oil concentrations, prevented the concentration-mortality curve calculation for these bees. The death cause may have been due to food starvation when the bees did not feed on the diet or by toxicity.

### Bee walking bioassays

**Figure 1:**
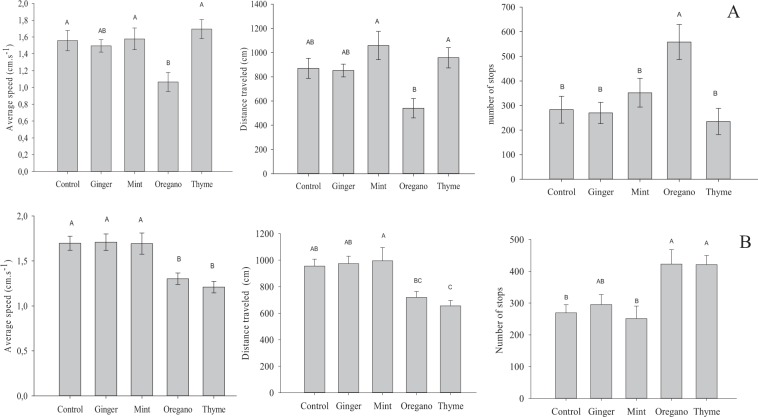
Routes and walking speeds of cumulative activities, distance traveled and number of stops by *Apis mellifera* (**A**) and *Trigona hyalinata* (**B**) (Hymenoptera: Apidae) of adults workers in contact with a surface treated in no-choice bioassays exposed with sublethal doses of four essential oils insecticides. Means followed by the same letter do not differ by the Tukey test (p < 0.05).

The distance traveled (F_1.69_ = 2.12, p < 0.05) and the walking speed (F_1.69_ = 2.72, p < 0.05) were lower and the number of stops higher F_1.69_ = 2.13, p < 0.05) for *A*. *mellifera* adults in areas treated with oregano essential oil. The values of these parameters were similar for the ginger, mint and thyme oils (Fig. 1). The walking speed (F_1.69_ = 3.62, p < 0.05) and the distance traveled (F_1.69_ = 3.02, p < 0.05) were lower and the number of stops higher (F_1.69_ = 1.87, p < 0.05) for *T*. *hyalinata* in areas treated with oregano and thyme oils than in the control (Fig. 1). Characteristic patterns of *A*. *mellifera* and *T*. *hyalinata* walking in fully treated areas showed lower agitation of these insects in treatments with oregano (Fig. 1).

**Figure 2:**
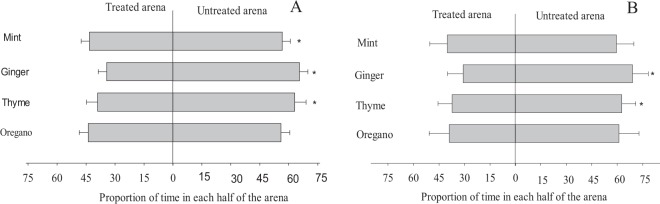
Time proportion (mean ± standard error) for *Apis mellifera* (**A**) and *Trigona hyalinata* (**B**) (Hymenoptera: Apidae) adults in half-treated or untreated area with each essential oil. *Asterisk indicates that the time proportion for the insect staying in this half of the area was greater than in the other according to the t test at P < 0.05.

Ginger (t_1.28_ = 4.89), mint (t_1.28_ = 2.17) and thyme (t_1.28_ = 2.82) essential oils reduced the time spent by *A*. *mellifera* on the treated half of the plate (Fig. 2). The time spent by *T*. *hyalinata* was lower in areas treated with ginger (t_1.28_ = 2.84) and thyme (t_1.28_ = 2.09) (Fig. 2). Characteristic patterns of *A*. *mellifera* and *T*. *hyalinata* walking in the partially treated areas showed that these bees did not avoid those contaminated by the oils (Fig. 2).

## Discussion

### Toxicity to *A. mellifera* and *T. hyalinata*

Chemical and biological control of pests require selective products for integrated pest management programs and insecticides should not impact pollinator insects, which are indispensable for the propagation of many cultivated and native plants^2,10,11^. The botanical product toxicity to insects such as natural enemies and pollinators should be evaluated due to the demand for organic food^29,30^. Insecticide stress in arthropods is not restricted to lethal effects, and sublethal ones are as important because the insects remain exposed to sublethal concentrations.

Differences in the toxicity between ginger, mint, oregano and thyme essential oils may be related to their penetration rates in *A*. *mellifera* and *T*. *hyalinata* as found for the lower *Trigona spinipes* Fabricius, 1793 (Hymenoptera: Apidae) survival at the concentrations of 3% to 7% of *Azadirachta indica* (Meliaceae)^29^. The penetration of these substances into the cuticle of the insects is proportional to their lipophilicity, with greater ease for lipophilic compounds similar to those of the cuticle^31,32^. Additionally, concentrations and activity of allelochemicals in botanical products, such as terpenes and insect and pathogen protective substances determine the toxicity of these products^22,23^.

Differences in the susceptibility between *A*. *mellifera* and *T*. *hyalinata* to ginger, mint, oregano and thyme essential oils differ from reports that the larger the bee, the lower the pesticide impact^24,33^. The vulnerability to insecticides depends on factors such as morphological changes (fat deposit levels), physiological (hemolymph pH) and presence of detoxifying enzymes (number of detoxifying genes in cytochrome P450)^34,35^. The penetration of substances and their translocation to the action target are also related to characteristics such as thickness, nature and lipophilic specific surface of insect cuticle^31,32^.

The lower slope (slope) of the concentration-mortality curve, for topical application than for contact, suggests differences in the susceptibility of *A*. *mellifera* and *T*. *hyalinata* adults according to the application mode. The curve decline may indicate the insecticide potency and the degree of genetic homogeneity involved in the tolerance, for example, due to the genes for enzyme detoxification^36^. The slope values of the curves are higher for insect populations with homogeneous responses, indicating that small variations in the dose of the product cause significant changes in mortality and increase the probability of selecting resistant individuals^37^. Insecticides with this response should be avoided at foraging times to reduce harmful effects on pollinators in the field.

The higher toxicity of oregano oil to *A*. *mellifera* by contact and topical application suggests caution in its use and the adoption of principles of ecological selectivity, applying them during low foraging and out-of-flowering times to minimize the impact on bees^38^. The major compound of botanical insecticides is generally responsible for its toxic action, isolated or synergistically with the others^22^. The higher toxicity of oregano oil can be attributed to its phenol compound (carvacrol), more toxic than ginger hydrocarbons (zingiberene) and alcohol in menthol^22^. The mode of action of terpenoids, also present in essential oils, in insects, differs. Monoterpenoid phenols usually have high insecticidal potential, and insect mortalit primarily through neurotoxic effects^39,40^. Carvacrol and thymol may inhibite the enzyme acetylcholinesterase, consequently leading to overstimulation of neurons in insects^39,40^. Oregano oil was the more toxic to *A*. *mellifera*, but with CLs higher for these pollinators than reported for the pests *Anticarsia gemmatalis* (Lepidoptera: Noctuidae) (LC_25_ = 0.13%)^14^, *Euproctis chrysorrhoea* (Lepidoptera: Lymantriidae) (LC_50_ = 0.05%)^22^, *Thaumetopoea wilkinsoni* Tams. (Lepidoptera: Thaumatopoeidae) (CL_50_ = 0.31%)^41^ and the citrus mealybug [*Planococcus citri* (Risso) (Hemiptera:Pseudococcidae)] (LC_25_ = 0.06%)^42^. Oregano oil is promising for integrated pest management with *Trichogramma pretiosum* (Riley) (Hymenoptera: Trichogrammatidae) an important biological control agent of agricultural and forest pests^43^. The oregano oils showed toxicity to the natural enemy *Chrysoperla externa* (Neuroptera: Chrysopidae) (LD_50_ < 0.2%), but it was relatively safe compared to natural pyrethrins, making it a potential candidate for selective and efficient botanical insecticide^44^. This information should be considered for the use of these oils. The intermediate impact of oregano oil on *A*. *mellifera* and *T*. *hyalinata* and their potential for pest control indicate the importance of application at the recommended doses and at times of lower pollinator activity.

The toxicity of thyme compared to the other oils, especially for *A*. *mellifera*, corroborates reports of a reduction in movement and wax production by this bee exposed to this product^19^. Concern regarding this extract is due to the thymol compound and used to control the ectoparasite mite *Varroa destructor* Anderson and Trueman (Acari: Varroidae) in *A*. *mellifera*^19,45^. This oil is used to control this mite, but it should be applied at suitable times (large infestations) and with the doses recommended to avoid or reduce the impact on the hive. The toxic concentration of this product to *T*. *hyalinata* and *A*. *mellifera* was higher than that recommended for the control of the pests *A*. *gemmatalis* (LC_25_ = 0,41%)^14^, *Choristoneura rosaceana* (Lepidoptera: Tortricidae) (LC_50_ = 0,56%), *Trichoplusia ni* (Lepidoptera: Noctuidae) (LC_50_ = 1,1%)^23^ and the citrus mealybug, *P. citri* (LC_25_ = 0.06%)^42^. The botanicals insecticides should be less toxic than synthetic insecticides, such as the thymol being 50 times less toxic to bees than the dimethoate^10^. The *T*. *vulgaris* oil also is promising for integrated pest management with *T*. *pretiosum*^43^. The natural insecticide azadirachtin was less toxic than the imidacloprid to *Partamona helleri* Friese, 1900 and *Scaptotrigona xanthotricha* Moure 1950 (Hymenoptera: Apidae) at field doses^46^.

The low toxicity of mint and ginger essential oils to *A*. *mellifera* and *T*. *hyalinata* adults may be due to the absence of susceptibility of these insects and/or the lower penetration rate of their metabolites in the bee cuticle^32,47^. These products can be used in pest control during flowering when the bees forage in the plants. In addition, the residual half-life of botanical insecticides is generally shorter in the field, reducing the exposure time of non-target organisms. The botanical substance toxicity to bees was in decreasing order of oxalic acid> oregano; thymol> menthol is similar to control^30^. The product Apilife Var^®^, used to control *V*. *destructor*, presents menthol in its composition, but this substance was not detected in the brain or body of bees or in the wax produced^19^. The low mint oil toxicity was also reported for *Apis* sp. larvae, LC_50_ = 382.8 μg/larva, while that of thyme and oregano was toxic to this insect with LC_50_ = 150.7 and 236.4 μg/larva, respectively^25^. Mint oil was effective in controlling *Anarta trifolii* (Hufnagel) (Lepidoptera: Noctuidae)^48^ and *Tetranychus cinnabarinus* (Boisduval) (Acarina: Tetranychidae)^49^ with lower LC_50_ (<0.2%) than those found for *A*. *mellifera* and *T*. *hyalinata*. Mentha piperita oil prevented the emergence of *Callosobruchus chinensis* (L.) (Coleoptera: Bruchidae) and *Musca domestica* (L.) (Diptera: Muscidae)^50^ and reduced the emergence of *Acanthoscelides obtectus* (Say) (Coleoptera: Bruchidae) by 32%^51^. Thus, mint oil can be a reasonable alternative to chemical pesticides to control these pests due to its efficiency and low pollinator toxicity. However, measures to avoid toxicity and contact with non-target organisms should be prioritized.

Changes in the color, taste and odor of the diet treated with the ginger, mint, oregano and thyme oils may have chased away and prevented the bees from feeding. Neem oil was toxic through contact to *A*. *mellifera* but had no effect on ingestion due to the anti-nutritive property of its active ingredient azadirachtin^52^. Formulations of insect repellent compounds for bees may be useful to prevent their contact with areas recently treated with toxic insecticides.

### Bee walking bioassays

Monoterpenoids can affect learning, memory and gene expression in the brain of the *A*. *mellifera*^53^. This indicates the importance of risk assessment for botanical products on the behavior of pollinators due to bees potentially showing variability in their sensitivity.

### Total treated area

The shorter distance traveled and the slower speed of *A*. *mellifera* adults in contact with oregano essential oils and *T*. *hyalinata* in contact with oregano and thyme oils shows a tendency of this bee to slow walking or stop^35^. Insect walking behavior is affected by the insecticides tested and the overall reduced activity observed with exposure may be an adaptive response leading to reduced exposure to toxic residues^54^. This may allow the insect to avoid or reduce contact with the insecticide and the amount of pesticide accumulated in the tarsus^54^. The high toxicity and residual effect of insecticides can reduce insect movement^55^.

### Partially treated area

The shorter time spent by *A*. *mellifera* in areas treated with ginger, mint and thyme and by *T*. *hyalinata* in those with ginger and thyme suggests a reduction in these parameters due to behavioral changes and irritability for these pollinators^12,35^. The irritable behavior from insecticides may be associated with a neurotoxic response after exposure to the causative agent^56^. This characteristic and lower toxicity may reduce the ginger and mint oil impact on bees in the field. However, insects may be encouraged to change behavior and leave the environment after contact with the treated surface^57^. The repellent effect of the oils, when the bee avoided treated areas, was not observed with oregano oil, although it is toxic for both species tested. This may be related to rapid oil intoxication, altering pollinator behavior that stopped more often and with a similar period in the two halves of the treated area. Insecticides with repellent effects decrease the number of bees foraging on flowers, which might lead not only to inadequate nectar and pollen gathering but also to deficient crop pollination.

Essential oils from plants with insecticidal activity and safety from natural enemies can play important roles in IPM programs. The organic food production is the main market niche of plant essential oils^57,58^, and they are an economically viable alternative for small farmers to control pests^59^. The compounds evaluated in our study can be considered through their direct use or by serving as precursors for the synthesis of new selective insecticide active ingredients. Important synthetic insecticides from different chemical groups used in agriculture were originated from plant metabolites, but these compounds should not be exempt from a risk assessment, including the evaluation of possible lethal and sublethal effects on non-target organisms. In addition, possible phytotoxic effects on crops of interest should be evaluated. Essential oils, especially ginger, mint and thyme, have potential as insecticide for insect pest management, as these substances are toxic to pests and have low toxicity to *A*. *mellifera* and *T*. *hyalinata*.

## Conclusions

Mint and ginger oils were the most selective for *A*. *mellifera* and *T*. *hyalinata* and, therefore, can be used for pest management.

Oregano and thyme oils were mainly toxic to *A*. *mellifera* and should be used with caution in pest control. *Trigona hyalinata* was more tolerant to ginger, mint, oregano and thyme oils than *A*. *mellifera*.

Contact in areas treated with sublethal doses of oregano and thyme oils reduced the distance traveled and the movement speed and increased the number of stops by *T*. *hyalinata*. Oregano was the only oil that had this effect on *A*. *mellifera*.

Essential oils did not repel *A*. *mellifera* or *T*. *hyalinata*, but those of ginger, mint and thyme reduced the time spent by *A*. *mellifera* in areas treated with sublethal doses. This time reduction was observed with ginger and thyme for *T*. *hyalinata*.
